# Study on the Mechanism of Interaction between Dipeptidyl Peptidase 4 and Inhibitory Peptides Based on Gaussian Accelerated Molecular Dynamic Simulation

**DOI:** 10.3390/ijms25020839

**Published:** 2024-01-10

**Authors:** Yuyang Liu, Wencheng Zhao, Yongxin Jiang, Shu Xing, Wannan Li

**Affiliations:** 1 Edmond H. Fischer Signal Transduction Laboratory, School of Life Sciences, Jilin University, Changchun 130012, China; liuyy1319@mails.jlu.edu.cn (Y.L.); wczhao16@mails.jlu.edu.cn (W.Z.); 2 Key Laboratory for Molecular Enzymology and Engineering of Ministry of Education, School of Life Sciences, Jilin University, Changchun 130012, China; jiangyx23@mails.jlu.edu.cn

**Keywords:** inhibitory peptide, dipeptidyl peptidase 4 (DPP4), Gaussian accelerated molecular dynamics simulation, conformational changes, MM-PBSA

## Abstract

Dipeptidyl peptidase 4 (DPP4) inhibitors can effectively inhibit the activity of DPP4, increasing the concentrations of glucagon-like peptide-1 (GLP-1) and glucose-dependent insulinotropic polypeptide (GIP), which allows for them to effectively contribute to the reduction of blood sugar levels. Leu-Pro-Ala-Val-Thr-Ile-Arg (LPAVTIR) and Leu-Pro-Pro-Glu-His-Asp-Trp-Arg (LPPEHDWR) were the two peptides with the strongest inhibitory activity against DPP4 selected from silkworm pupa proteins. In this study, four systems were established: Apo (ligand-free DPP4), IPI (IPI-bound DPP4), LPAVTIR (LPAVTIR-bound DPP4), LPPEHDWR (LPPEHDWR-bound DPP4), and Gaussian accelerated molecular dynamic (GaMD) simulation was conducted to investigate the mechanism of action of two inhibitory peptides binding to DPP4. Our study revealed that the LPAVTIR peptide possessed a more stable structure and exhibited a tighter binding to the Ser630 active site in DPP4, thus exhibiting a favorable competitive inhibition effect. In contrast, the LPPEHDWR peptide caused the horizontal α-helix (residues 201–215) composed of Glu205 and Glu206 residues in DPP4 to disappear. The spatial arrangement of active sites Ser630 relative to Glu205 and Glu206 was disrupted, resulting in enzyme inactivation. Moreover, the size of the substrate channel and cavity volume was significantly reduced after the binding of the inhibitory peptide to the protein, which was an important factor in the inhibition of the enzyme activity. A similar effect was also found from IPI (our positive control). By stabilizing the active site of DPP4, the IPI peptide induced the disappearance of the horizontal α-helix and a notable reduction in the active cavity volume. In conclusion, our study provided a solid theoretical foundation for the inhibitory mechanisms of IPI, LPAVTIR, and LPPEHDWR on DPP4, offering valuable insights for advancing the development of drug targets for type 2 diabetes.

## 1. Introduction

Type 2 diabetes accounts for nearly 90% of the estimated 537 million cases of diabetes worldwide and is the most common type of diabetes [1]. It is characterized by the development of insulin resistance, which leads to pancreatic beta cell failure and ultimately to blood sugar dysregulation [2]. The main risk of diabetes is that high levels of blood sugar over a long period can lead to several complications, such as blindness, nerve damage, and renal failure [3].

Glucagon-like peptide-1 (GLP-1) and glucose-dependent insulinotropic polypeptide (GIP) are enteric-derived peptide hormones released by small intestinal enteroendocrine cells (EECs). They play a crucial role in maintaining glucose homeostasis by stimulating insulin secretion from pancreatic β-cells and inhibiting the release of glucagon [4]. Dipeptidyl peptidase 4 (DPP4), a highly specific serine protease, selectively cleaves peptides or dipeptides featuring alanine or proline in the penultimate position at the N-terminal of a hormone, resulting in the inactivation of the peptide. DPP4 plays a pivotal role in degrading GLP-1 and GIP, thus hindering their ability to exert hypoglycemic effects [5]. 

Dipeptidyl peptidase 4 (DPP4) inhibitors enhance the body’s ability to control its blood glucose by increasing the activity level of the hormone glucagon in the body. Their mechanism of action is different from any of the existing oral hypoglycemic agents. They control elevated blood glucose by triggering insulin secretion from the pancreas, inhibiting glucagon secretion, and signaling the liver to reduce glucose production [6,7].

Unfortunately, the various synthetic drugs used to treat type II diabetes (DPP4 inhibitors) typically cause more serious side effects [8], gastrointestinal adverse reactions [9], allergic reactions [10], skin-related side effects [11], and musculoskeletal disorders. However, DPP4 inhibitors have been found in foods, including milk, fish, wheat gluten, beans, and eggs, which are all natural protein sources, and their proteins may be degraded and release various DPP4 inhibitory peptides [12,13,14,15,16].

Silkworm pupae (Bombyx mori) are byproducts of cocoon extraction and constitute 60% of the mass of dried cocoons. Bombyx mori is rich in protein, presenting a high-quality natural protein. The development of bioactive peptides is a good direction for the application of silkworm pupa proteins [17]. Leu-Pro-Ala-Val-Thr-Ile-Arg (LPAVTIR) and Leu-Pro-Pro-Glu-His-Asp-Trp-Arg (LPPEHDWR) were identified as the two peptides with the strongest inhibitory activity of DPP4, with IC_50_ values of 192.47 μM and 261.17 μM, respectively [18]. The enzyme kinetic data suggest that these two peptides have a mixed-type DPP4 inhibition pattern [18]. However, the precise mechanism underlying the inhibition of DPP4 by these two peptides remains unclear.

Gaussian accelerated molecular dynamic (GaMD) simulation is a robust computational technique, which provides simultaneous unconstrained, enhanced sampling and free energy calculations of biomolecules [19]. GaMD simulation has a wide range of applications in biological systems, including studying protein–ligand binding [20], studying the effects of inhibitors on protein structure [21], identifying binding sites [22], and elucidating drug pathways [23]. Beforehand, GaMD simulations were employed to investigate the inhibition mechanism of enzymes related to peptide inhibition [24]. Ile-Pro-Ile (IPI) has been reported to be the most potent DPP-IV inhibitory peptide (IC_50_ = 5 μM) [25]. Therefore, we selected IPI as a positive control.

In this study, we performed 500 ns GaMD simulation of four systems, Apo (ligand-free DPP4), LPAVTIR (LPAVTIR-bound DPP4), LPPEHDWR (LPPEHDWR-bound DPP4), to deeply investigate the molecular mechanisms of the inhibition of the inhibitory peptides. The results of the GaMD simulation was analyzed to obtain the sites of interaction between the inhibitory peptide and DPP4, and the microscopic changes of protein conformation after binding, which will provide ideas for the development of related drugs.

## 2. Results and Discussion

### 2.1. The Binding Mode of Inhibitors to DPP4

**Figure 1:**
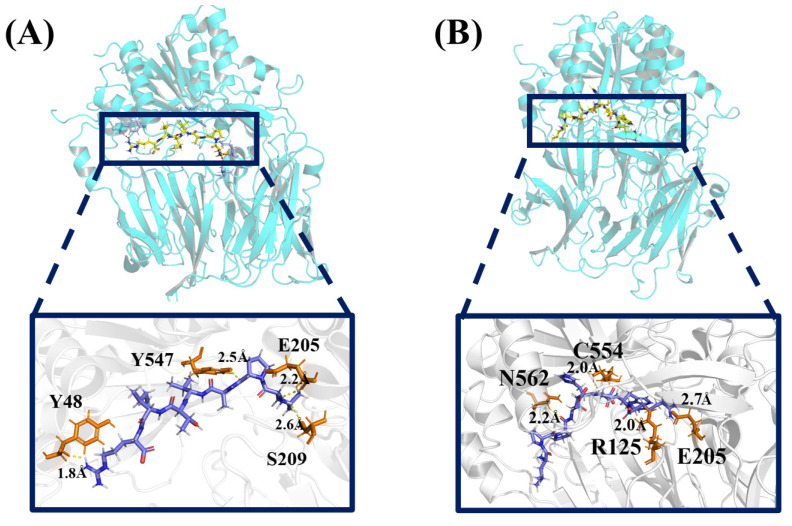
(**A**) The hydrogen bonds between the inhibitory peptide LPAVTIR and DPP4. (**B**) The hydrogen bonds between the inhibitory peptide LPPEHDWR and DPP4.

DPP4 is a dimer, and each subunit consists of two domains, an α/β-hydrolase domain and an eight-bladed β-propeller domain. The serine-protease active triad includes Ser630, Asn708, and His740. The blue structures in the figures are inhibitory peptides, and the orange parts are residues that hydrogen bond with inhibitory peptides. The molecular docking results of DPP4 and the two inhibitory peptides are shown in Figure 1A,B. Inhibitory peptide LPAVTIR forms a hydrogen bond network with Tyr48, Ser209, Glu205, Tyr547 of DPP4; carbon–hydrogen bonds with Glu205, Glu206, Gly741; amide–pi stacked interactions with His748; and pi–alkyl interactions with Phe357, Trp629, Tyr666, His748, Tyr752 (Figure S1). This docking result indicates that LPAVTIR binds to the cavity region involved in protein-catalyzed core interactions. Inhibitory peptide LPPEHDWR forms hydrogen bonds with Asn562 of DPP4 and with Lys554; carbon–hydrogen bonds with Glu205, Gln553; alkyl interactions with Tyr48, Arg560, and Ala564; and pi–anion interactions with Ala545 (Figure S2), which indicates that LPPEHDWR is also involved in protein-catalyzed core interactions. According to the docking results, it is observed that both inhibitory peptides bind to the active site of DPP4. By comparing the other docking sites and evaluating the binding energies, it can be inferred that both complexes have a stable structure, indicating their suitability as initial conformations for MD simulation.

### 2.2. Structural Stability and Dynamic Properties of the Four Systems

**Figure 2:**
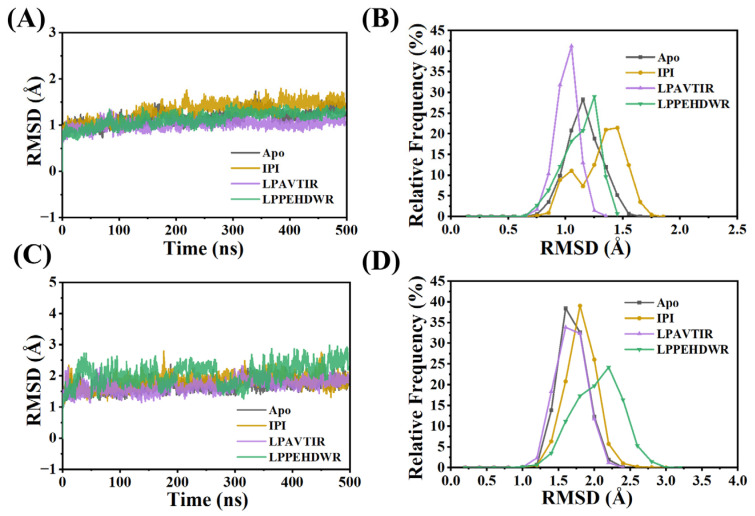
(**A**) Variation of RMSD values of the α/β-hydrolase domains of the four systems. (**B**) Relative frequency distribution of RMSD of the α/β-hydrolase domains of the four systems. (**C**) Variation of RMSD values of the eight-bladed β-propeller domain of the four systems. (**D**) Relative frequency distribution of RMSD of the eight-bladed β-propeller domain of the four systems.

DPP4 comprises an α/β hydrolase structural domain and an eight-bladed β propeller structural domain; we analyzed these two domains separately in this study. To evaluate the stability of the simulation, the root mean square deviation (RMSD) of the CA atoms was calculated (Figure 2). In the results, the RSMD values of the α/β-hydrolase domains of the four systems are all stable around 1 Å, which means that the part of this domain has no obvious fluctuation.

In the eight-bladed β-propeller domain, the mean RMSD values for the four systems (Apo, IPI, LPAVTIR, and LPPEHDWR) were 1.69 Å, 1.81 Å, 1.67 Å, and 2.05 Å, respectively, with standard deviations of 0.19 Å, 0.21 Å, 0.20 Å, and 0.32 Å. The LPPEHDWR system exhibits an average RSMD of 2.05 Å, a value considerably higher than observed in the other three systems. This suggests that the LPPEHDWR system experiences significant fluctuations and may have undergone structural changes. we observed that the RMSDs in each MD trajectory reached equilibrium, indicating that all the systems studied were stable and could be used for a subsequent analysis.

**Figure 3:**
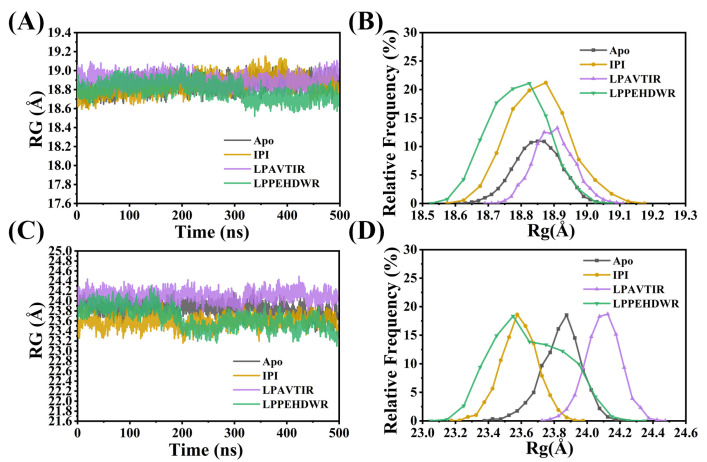
(**A**) Variation of RG values of the α/β-hydrolase domains of the four systems. (**B**) Relative frequency distribution of RG of the α/β-hydrolase domains of the four systems. (**C**) Variation of RG values of the eight-bladed β-propeller domain of the four systems. (**D**) Relative frequency distribution of RG of the eight-bladed β-propeller domain of the four systems.

The radius of gyration (R_g_) was employed to assess changes in protein compactness within the simulation. In the Apo and LPAVTIR systems, the R_g_ values of the α/β hydrolase structural domains fluctuate around 18.85 Å, while the R_g_ values for LPPEHDWR have a slight decline during the simulation (Figure 3A,B). In the eight-bladed β-propeller domain, the mean R_g_ values for the four systems (Apo, IPI, LPAVTIR, and LPPEHDWR) were 23.84 Å, 23.59 Å, 24.10 Å, and 23.65 Å, respectively, with standard deviations of 0.12 Å, 0.11 Å, 0.10 Å, and 0.21 Å. R_g_ for LPPEHDWR significantly decreased accompanied by notable fluctuations in standard deviation, which suggests that the binding of LPPEHDWR to DPP4 enhances protein compactness (Figure 3C,D).

**Figure 4:**
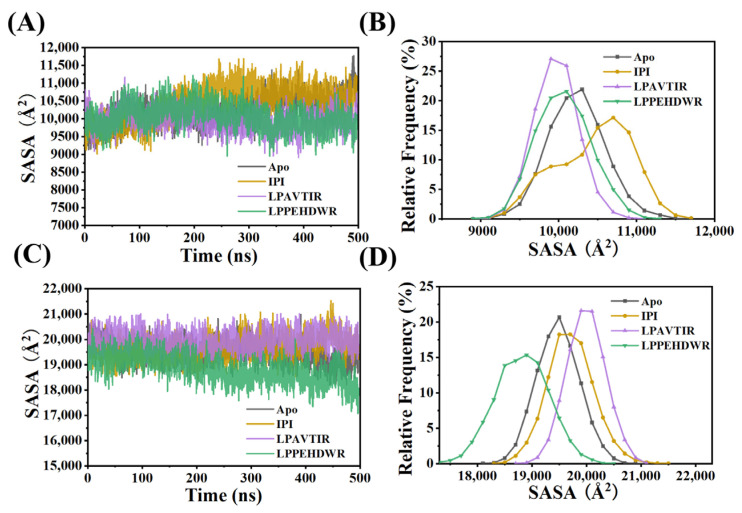
(**A**) Variation of SASA values of the α/β-hydrolase domains of the four systems. (**B**) Relative frequency distribution of SASA of the α/β-hydrolase domains of the four systems. (**C**) Variation of SASA values of the eight-bladed β-propeller domain of the four systems. (**D**) Relative frequency distribution of SASA of the eight-bladed β-propeller domain of the four systems.

The soluble surface area (SASA) was used to predict the number of residues in the exposed regions (surface) and the hydrophobic core (buried) of the protein. The SASA values of the systems during the 500 ns MD are depicted in Figure 4. In the case of the α/β hydrolase structural domain, the SASA values for the four systems were slightly lower (Figure 4A,B), which can be attributed to the binding of the hydrophobic inhibitor in this region, indicating strong binding. In the eight-bladed β-propeller domain, the mean SASA values for the four systems (Apo, IPI, LPAVTIR, and LPPEHDWR) were 19,477.78 Å^2^, 19,709.62 Å^2^, 19,989.47 Å^2^, and 18,815.23 Å^2^, respectively, with standard deviations of 386.35 Å^2^, 422.75 Å^2^, 350.50 Å^2^, and 489.98 Å^2^. In the LPPEHDWR system, the SASA was markedly lower, measuring 18,815.23 Å^2^, significantly lower than the average value of 19,477.78 Å^2^ in the Apo system (Figure 4C,D). These SASA results were consistent with the changes observed in R_g_, suggesting that the binding of the inhibitory peptide LPPEHDWR may have induced conformational changes in the protein.

In conclusion, all systems remained stable after the 500 ns MD simulation and can be utilized for further investigations.

### 2.3. Flexibility Analysis of DPP4 Protein

**Figure 5:**
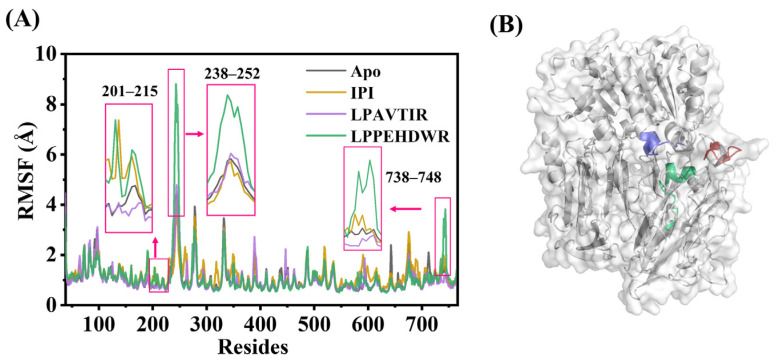
(**A**) The RMSF diagrams of four systems. (**B**) Residue locations in the protein: green represents residues 201–215, red corresponds to residues 238–252, and blue indicates residues 738–748.

The root mean square fluctuation (RMSF) values can be used to assess the flexibility of amino acid residues. Figure 5A illustrates the RMSF values of C_α_ atoms in DPP4 for the four systems. By combining the data, we identified regions with significant differences in the fluctuation of amino acid residues among the four systems, highlighted as the red region in the figure. These regions are residues 201–215, residues 238–252, and residues 738–748, as shown in Figure 5B. Residues 201–215 form horizontally helical structures containing Glu205 and Glu206, crucial for the spatial arrangement of the active site Ser630, influencing pre-peptide cleavage. Residues 738–748 also interact closely with the active site. Residues 238–252 constitute an extended arm and exhibit the highest degree of fluctuation, potentially playing a role in protein volume changes. Given their close relationship with protein activity, these three regions were selected for further analysis.

### 2.4. Comparison of the Conformational Changes of the Four Systems

**Figure 6:**
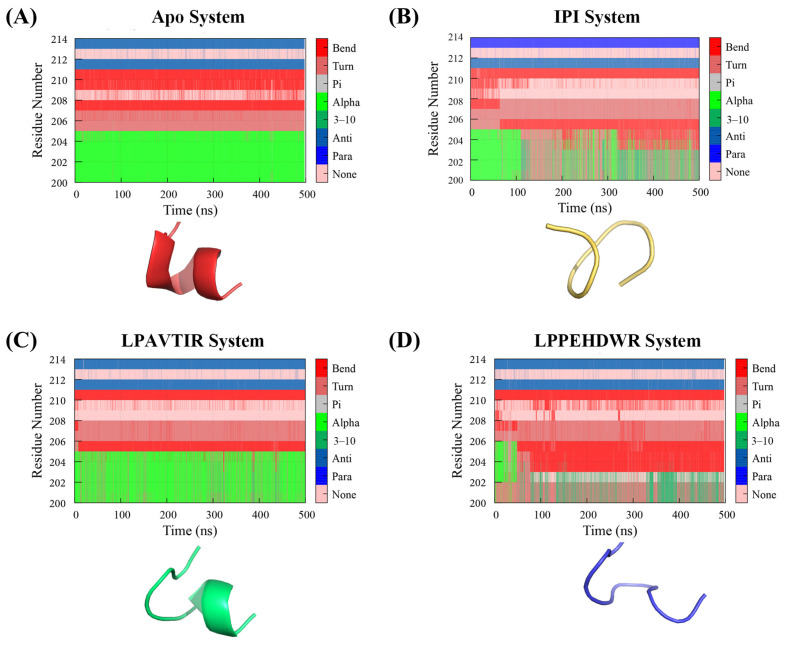
(**A**) The secondary structure changes’ probability and the corresponding 3D structure changes of the Apo system in residues 201–215. (**B**) The secondary structure changes’ probability and the corresponding 3D structure changes of the IPI system in residues 201–215. (**C**) The secondary structure changes’ probability and the corresponding 3D structure changes of the LPAVTIR system in residues 201–215. (**D**) The secondary structure changes’ probability and the corresponding 3D structure changes of the LPPEHDWR system in residues 201–215.

Protein secondary structure studies play a crucial role in molecular dynamics simulation. Figure 6 illustrates the changes in secondary structure for residues 201–215. Likewise, Figure S3 displays the three-dimensional structural variations of residues 738–748 conformations, with red indicating Apo, yellow indicating IPI, green representing the LPAVTIR system, and blue representing the LPPEHDWR system. It can be seen that the α-helix of residues 738–748 disappears around 300 ns of the simulation in the LPPEHDWR system, and the corresponding structural changes are shown in Figure S3.

For a clearer understanding of the α-helix alterations in residues 201–215 during the simulation, please consult Figure 6, where their corresponding structural changes are illustrated. Additionally, Table 1 presents the probability of α-helices’ occurrence throughout the simulation. Specifically, the residue 201–215 reveals an α-helix appearance probability of 99.78% in the Apo system, 38.41% in the IPI system, 80.34% in the LPAVTIR system, and 6.68% in the LPPEHDWR system. Notably, the LPAVTIR system modestly reduces the α-helix, the IPI system significantly diminishes the α-helix, and the LPPEHDWR system almost eliminates this helical segment. The spatial arrangement of the active site Ser630 in relation to Glu205 and Glu206 is a crucial feature influencing the cleavage of pre-peptides. Consequently, the disappearance of the horizontal α-helix leads to the disruption of the spatial arrangement between site Ser630 and Glu205/Glu206, affecting the enzymatic activity of DPP4. This particular structural change is believed to contribute to the inhibition of DPP4.

### 2.5. Dynamics Cross-Correlation Matrix and Principle Component Analysis

**Figure 7:**
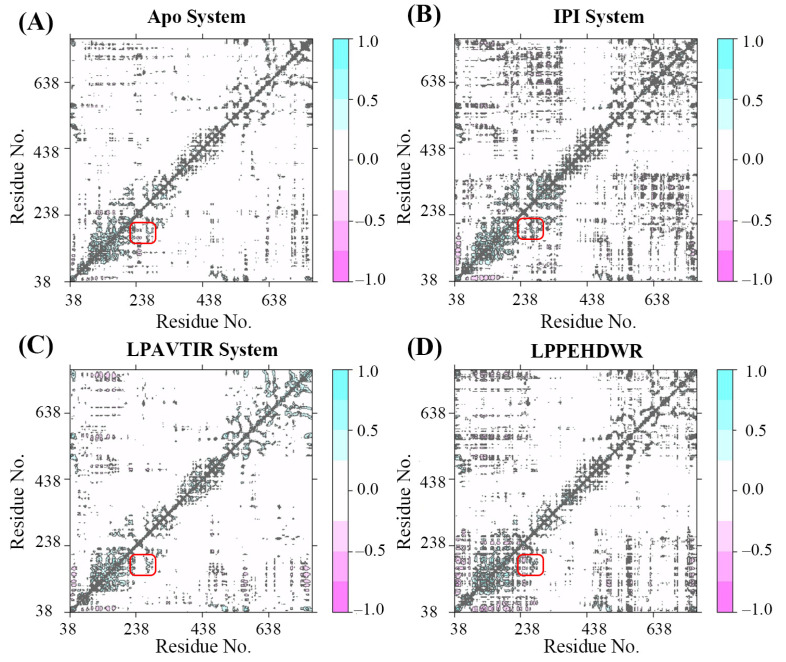
(**A**) The Dynamical Cross-Correlation Matrix diagrams of Apo. (**B**) The Dynamical Cross-Correlation Matrix diagrams of IPI. (**C**) The Dynamical Cross-Correlation Matrix diagrams of LPAVTIR. (**D**) The Dynamical Cross-Correlation Matrix diagrams of LPPEHDWR.

The dynamical cross-correlation matrix (DCCM) analyses of all C_α_ atoms are presented in Figure 7A–D. Positive regions are depicted in cyan, indicating correlation motions between residue C_α_ atoms, while negative regions are shown in pink, representing anti-correlation motions. In particular, the red rectangle within the figure highlights the interaction between residues 238–252 and the active site.

Comparatively, the IPI and LPPEHDWR systems exhibit a darker color than the Apo system, suggesting that the former experiences greater fluctuations during the MD simulation, leading to significant structural changes. Notably, the negative correlation observed between motions of residues 238–252 and 201–215 implies that the former undergoes negatively correlated motions about the active cavity position. This suggests that the extended arm region might approach the active cavity and assume a closed state.

**Figure 8:**
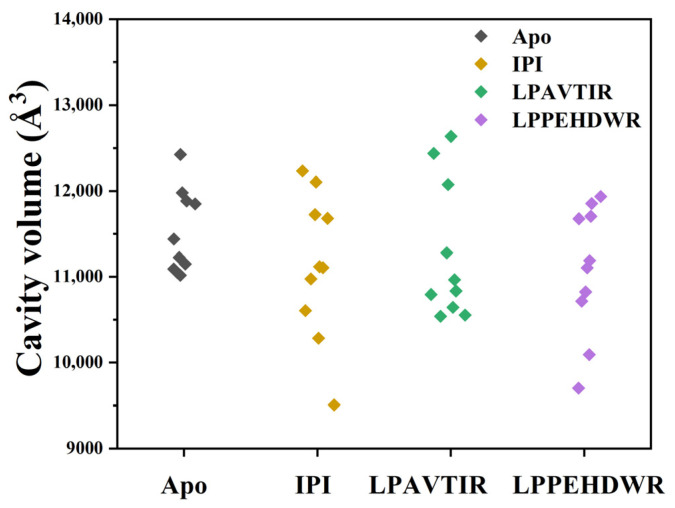
Active site volume analysis for the four systems.

The result of the simulation was subjected to cluster analysis, resulting in 10 distinct classes. To investigate whether inhibitory peptide binding affected the conformational change in the active site of DPP4, the active site cavity volume of 10 average protein structures obtained from a cluster analysis for four systems were calculated using the online server CASTp3.0 [26], and the results were presented in Table 2 and Figure 8. Compared to the Apo group, the other three systems showed a decrease in active cavity volume, with greater reductions in the IPI and LPPEHDWR systems. The measured cavity volumes of the Apo, IPI, LPAVTIR, and LPPEHDWR systems were 11,510.64 Å^3^, 11,133.39 Å^3^, 11,275.55 Å^3^, and 11,079.26 Å^3^, respectively, with standard deviations of 489.73 Å^3^, 847.61 Å^3^, 805.07 Å^3^, and 756.91 Å^3^. The LPPEHDWR system had a significantly smaller volume and smaller variance of the active cavity of the DPP4 protein compared to the other systems.

**Figure 9:**
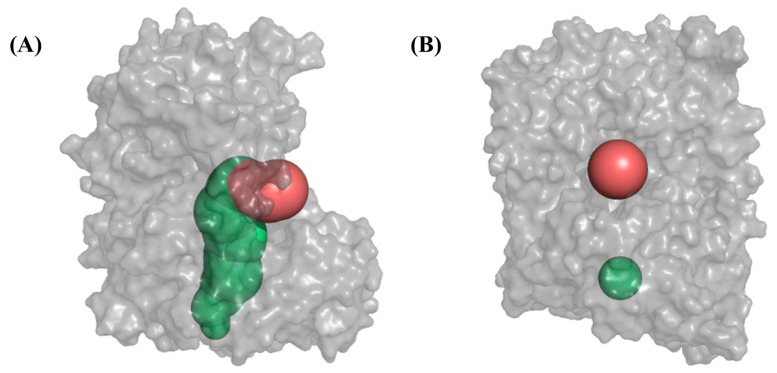
(**A**) The side (red) and bottom (green) openings of DPP4. (**B**) The minimum diameters of side (red) and bottom (green) openings of DPP4.

Next, representative structures were selected from the clustered results to measure the diameters of the two channels by CAVER3.0 [27], and these two channels are shown schematically in Figure 9. The diameter is the minimum of the protein channel. The measurements are presented in Table 3, revealing that the diameter of the bottom channel of the three systems remained relatively unchanged. Compared to the Apo system, the other three systems exhibited a decrease in the diameter of the side channel, with the IPI and LPPEHDWR systems experiencing a more pronounced reduction. The size of the channel diameter plays a crucial role in substrate entry and is an important factor in peptide inhibition.

Subsequently, a principal component analysis was performed on the systems. The two largest eigenvalues, PC1 and PC2, were used as reaction coordinates to calculate the relative Gibbs free energy and generate a Gibbs free energy surface (FEL). The FEL provides valuable information about different conformational states and reveals energy barriers between different conformations or states in the protein.

**Figure 10:**
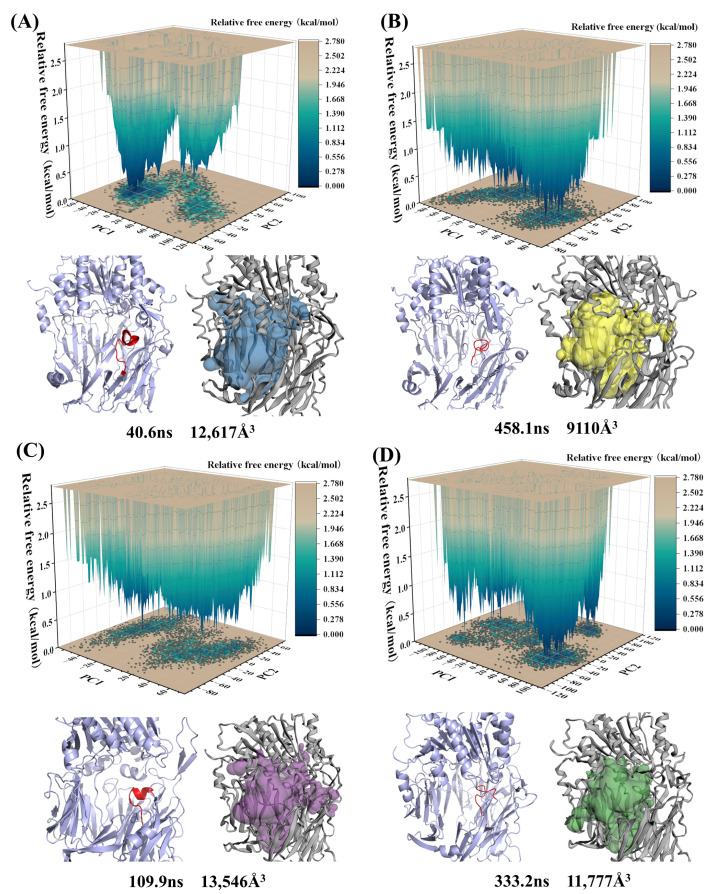
The free energy landscape for the following four systems: (**A**) Apo, (**B**) IPI, (**C**) LPAVTIR, and (**D**) LPPEHDWR. The low-energy conformations are displayed, highlighting changes in the 3D structure of residues 201–215 and protein solvent volumes.

Figure 10 illustrates the free energy surface diagram of the four systems. The low-energy conformations are displayed, highlighting changes in the 3D structure of residues 201–215 and protein solvent volumes. In the Apo system, the global energy minimum (PC1: −34.18, PC2: −10.14) was selected as the reference for analysis (Figure 10A). The Apo system represents the structure at the 40.6 ns time point, featuring a solvent volume of 12,617 Å^3^ and exhibiting an α-helix in residues 201–215. In Figure 10B, the free energy surface diagram of the IPI system is presented. Following comparative calculations, the global energy minimum (PC1: −41.70, PC2: 10.27) was chosen as the reference for the analysis. The corresponding conformation occurred at 458.1 ns, with a solvent volume of 9110 Å^3^. It can be seen that the solvent volume is significantly reduced and the α-helix of residues 201–215 disappears.

In Figure 10C, the free energy surface diagram of the LPAVTIR system is presented. Following comparative calculations, the global energy minimum (PC1: −42.68, PC2: 1.26) was chosen as the reference for the analysis. The corresponding conformation occurred at 109.9 ns, with a solvent volume of 13,546 Å^3^. Figure 10D displays the free energy surface diagram of the LPPEHDWR system. After comparative calculations, the global energy minimum (PC1: 64.01, PC2: −27.50) was selected as the reference for the analysis. The corresponding conformation was observed at 333.2 ns, with a solvent volume of 11,777 Å^3^. The results indicate a substantial reduction in protein solvent volume and the disappearance of the α-helix in residues 201–215 in the LPPEHDWR system. These factors are crucial for the inhibition of DPP4.

By comparison, it can be seen that the solvent volume values of both IPI and LPPEHDWR are significantly reduced and the α-helices of residues 201–215 disappear, which is in agreement with the results obtained previously.

### 2.6. Analysis of the Interaction between DPP4 and Dipeptide Inhibitors

**Figure 11:**
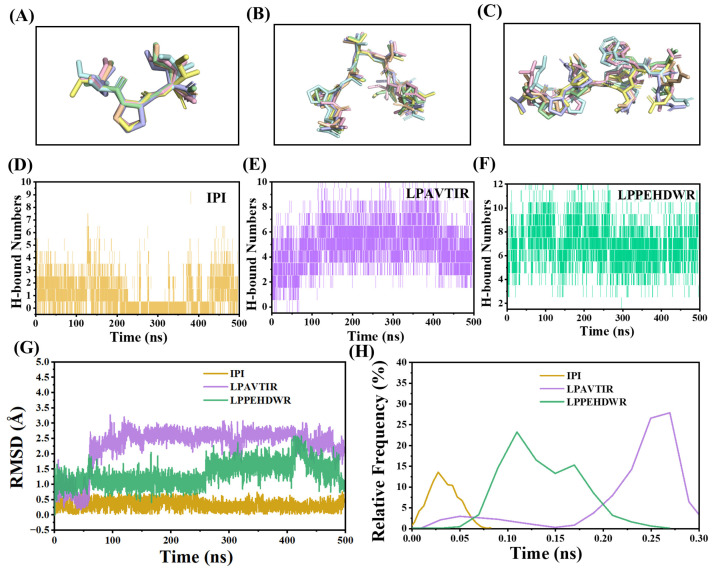
Ligand poses of six superimposed structures over 500 ns: (**A**) IPI, (**B**) LPAVTIR, and (**C**) LPPEHDWR. Evolution of the number of hydrogen bonds formed between DPP4 and peptides during molecular dynamic simulation: (**D**) IPI, (**E**) LPAVTIR, and (**F**) LPPEHDWR. (**G**) Time evolution of the RMSDs and (**H**) corresponding frequencies of IPI, LPAVTIR, and LPPEHDWR.

Through a cluster analysis of the 500 ns kinetic simulation, the patterns of the six average ligands during the MD simulation were obtained. The structures of IPI, LPAVTIR, and LPPEHDWR are shown in Figure 11A–C, respectively. It can be observed that the structure of IPI and LPAVTIR remains more stable throughout the simulation compared to LPPEHDWR. Figure 11D–F depict the variations in the number of hydrogen bonds formed by the inhibitory peptides IPI, LPAVTIR, and LPPEHDWR with DPP4 during the simulation, respectively. Due to the differing peptide lengths, LPAVTIR and LPPEHDWR form more hydrogen bonds with the protein. However, the number of hydrogen bonds for IPI remains relatively stable. This observation can also be indicative of the stability of the inhibitory peptide and its binding to the protein.

The changes in the root mean square deviation (RMSD) values and their corresponding relative frequency distributions are depicted in Figure 11G,H. The RMSD values of LPAVTIR are predominantly distributed around 2.5 Å, indicating relatively smaller fluctuations and increased stability as the simulation progresses. On the other hand, the degree of fluctuation in the RMSD value of LPPEHDWR is relatively large, indicating that it moves more vigorously during the simulation process. The IPI consistently stabilized around 0.34 Å, suggesting the enduring stability and tight binding of the IPI structure to the protein’s active site without deviation.

**Figure 12:**
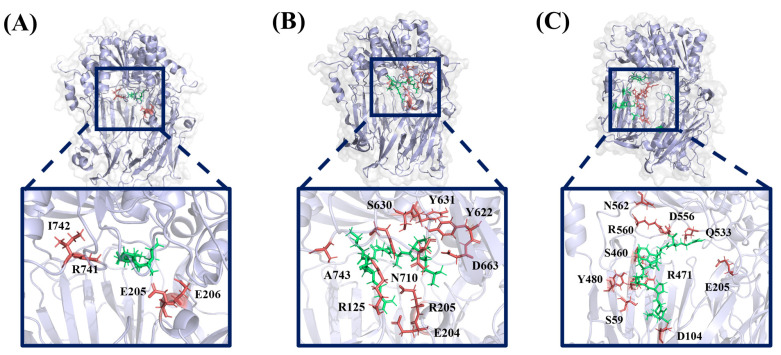
Binding pocket of (**A**) IPI, (**B**) LPAVTIR, and (**C**) LPPEHDWR systems during molecular dynamic simulation. Residues that interacted with the ligands are shown in red and inhibitory peptides are shown in green.

To further investigate the detailed interactions between the proteins and the inhibitors, representative structures obtained from the cluster analysis are utilized (Figure 12). In Figure 12, the major hydrogen bonding interacting residues during the simulation are highlighted in red. Inhibitory peptide IPI interacts with DPP4 through hydrogen bonds involving residues Glu205, Glu206, Arg741, and Ile742 (Figure 12A). LPAVTIR forms hydrogen bonds mainly with residues Ser630, Arg205, Asn710, Ala743, Tyr631, Tyr622, Asp663, and Arg125 (Figure 12B). In contrast, LPPEHDWR interacts with DPP4 using residues Ser460, Arg560, Asn562, Tyr480, Ser59, Asp104, Gln533, Asp556, Glu205, and Asp104 but does not bind to the active pocket (Figure 12C). Upon comparison, it is evident that inhibitory peptide LPPEHDWR does not bind to the active pocket, while inhibitory peptide LPAVTIR is similar in that it predominantly occupies the substrate-binding site in the active pocket, which is a key factor contributing to its inhibitory activity.

The results of MM-PBSA calculations are summarized in Table 4. The LPAVTIR-bound DPP4 complex exhibits a binding free energy of −33.74 ± 0.79 KJ/mol, and the LPPEHDWR-bound DPP4 complex displays a binding free energy of −32.91 ± 0.72 KJ/mol. These results indicate that LPAVTIR binds more strongly to DPP4 compared to LPPEHDWR. Overall, the tight and strong binding of LPAVTIR to the active site is consistent with its competitive inhibition. The free energy of IPI binding to DPP4 was −11.20 ± 0.72. While its binding ability was not as high as that of LPAVTIR and LPPEHDWR, IPI demonstrated stable binding to the active site of DPP4. This stability is a key factor contributing to the robust inhibition exhibited by IPI.

## 3. Materials and Methods

### 3.1. System Preparation

The three-dimensional structure of the DPP4 protein and inhibitor–ligand complex was obtained by searching the RCSB PDB database [28] (PDB ID: 2RIP [29]). The obtained pdb structure was processed using Pymol 2.4.0, an open source software, removing redundant water molecules, deleting small ligand inhibitor molecules, and saving it as a new pdb file of only the receptor protein DPP4.

HPEPDOCK 2.0 [30] can fully account for peptide flexibility by generating a large number of peptide conformations (up to 1000). Instead of constructing the peptide structure and optimizing the energy by ourselves, we used the sequence module, and the docking of the peptide to the DPP4 protein was performed using the HPEPDOCK 2.0 online website. Receptor proteins were selected from our processed pdb files and uploaded, and ligands were pasted in FASTA format at sequence. After conducting separate docking experiments of the DPP4 protein with IPI, LPAVTIR, and LPPEHDWR peptides, the model exhibiting the lowest binding energy was carefully selected for subsequent visualization and analysis of the interactions. The systems studied are named as follows IPI for IPI-bound DPP4, LPAVTIR for LPAVTIR-bound DPP4, LPPEHDWR for LPPEHDWR-bound DPP4, and Apo for ligand-free DPP4 protein.

### 3.2. Conventional Molecular Dynamic Simulation

Conventional molecular dynamic simulation (cMD) of the three model systems was performed using the pmemd.cuda module of AMBER 16 [31]. Before the simulation, force field parameters for the proteins and peptides were generated using the Leap module embedded in AMBER 16, both using the ff14SB force field [32]. Each system was then dissolved in an octahedral box using the TIP3P [33] water model. To prevent edge effects, periodic boundary conditions (PBC) were applied to the four systems. The distance between the solute surface and the box was set to 15 Å. An appropriate amount of antagonist ion (Na+) was added to neutralize the systems. All bonds involving hydrogen atoms were constrained using the SHAKE algorithm [34]. Non-bonded electrostatic interactions were handled using the particle mesh Ewald (PME) algorithm [35] with an intercept of 10 Å. In the minimization phase, the steepest descent algorithm and the conjugate gradient algorithm were used, each performed for 5000 steps. Then, the three models were gradually heated under the NVT set to 300 K. Finally, the 50 ns simulation of the equilibrium of the system was performed under the NPT set. The time step of the whole simulation was 2 fs.

### 3.3. Gaussian Accelerated Molecular Dynamic Simulation

The initial structures used for GaMD simulation were derived from the equilibrium structure of cMD simulation. In the GaMD method, the harmonic boost potential was added to reduce the energy barrier by smoothing the potential energy surface, thus accelerating transitions between different conformational states for enhanced sampling [36]. Here, the added lifting potential followed the Gaussian distribution so that the original potential energy surface could be easily recovered. In addition, GaMD has the benefit of not requiring any predefined reaction coordinates or collective variables (CVs). Therefore, this enhanced simulation method is excellently suited for studying the dynamics of complex biological systems. In this study, we applied dual potential boost [37] to the GaMD simulation. The dual potential parameters were determined from a previous 50 ns cMD simulation. Then, a 50 ns GaMD simulation was performed. Finally, 500 ns GaMD simulations were performed in the NVT ensemble, with coordinates saved every 10 ps.

### 3.4. Trajectory Analysis

All analyses, including RMSD, RMSF, Rg, SASA, and DCCM, were computed using Amber16′s Cpptraj module [38]. Principal component analysis (PCA) [39] was also calculated using Cpptraj. This is a widely used dimensionality reduction method to characterize the coordinated motion of an entire protein. Free energy mapping (FEL) is commonly used to find major conformations and their corresponding potentials.

### 3.5. MM-PBSA Calculations

The accurate calculation of protein–protein binding free energy is of great importance in biological and medical science [40]. This work used the molecular mechanics/Poisson–Boltzmann surface area (MM/PBSA) method to explore the ligands’ binding affinity to DPP4 [41,42]. The binding free energy (∆G_bind_) can be expressed by the following equations.

∆G_bind_ = ∆H − T∆S (1)

∆H = ∆E_MM_ + ∆G_sol_ (2)

∆E_MM_ = ∆E_ele_ + ∆E_vdW_ + ∆E_int_ (3)

∆G_sol_ = ∆G_pol_ + ∆G_nonpol_ (4)

where ∆E_MM_ and ∆G_sol_, represent the gas-phase molecular mechanical energy change and the solvation free energy change, respectively. Because there is little conformational change before and after receptor–ligand binding, this contribution of T∆S can be canceled out in the calculation of the difference [43]. ∆E_MM_ includes three terms calculated using molecular mechanics (MM): the covalent energy change (∆E_int_), the electrostatic energy change (∆E_ele_), and the van der Waals energy (∆E_vdW_). The ∆E_int_ consists of changes in the bond terms, angle terms, and torsion terms, respectively [44].

In this investigation, the conformational structures of the protein–ligand complex, as well as those of the individual protein and ligand, were derived from a sole MD trajectory, wherein the protein–ligand structure was treated as a rigid entity. Hence, the ∆E_int_ between the complex and the isolated components might counterbalance each other, as this energy term was computed using the identical MD simulated trajectory. Furthermore, only the ∆Eele and ∆EvdW components of Equation (3) were investigated in the subsequent analysis. ∆G_sol_ was used to indicate the sum of the polar solvation-free energy (∆G_pol_) and non-polar solvation-free energy (∆G_nonpol_). ∆G_pol_ was determined by solving the linearized Poisson–Boltzmann equation using the PBSA program in the AMBER 16 suite [31].

Subsequently, a total of 400 snapshots were extracted from the final trajectory spanning 100–500 ns at 10-frame intervals for MM/PBSA calculation [45].

## 4. Conclusions

Through 500 ns GaMD simulation of four systems, we have elucidated the inhibition mechanisms of three peptides on the DPP4 enzyme. Specifically, the LPAVTIR peptide demonstrated a more stable structure and tighter binding to the Ser630 active site of DPP4, resulting in superior competitive inhibition. In contrast, the LPPEHDWR peptide caused the disappearance of the horizontal α-helix (residues 201–215), comprising Glu205 and Glu206, in the DPP4 enzyme. This disruption of the spatial arrangement between the active site Ser630 and Glu205/Glu206 led to enzyme inactivation. Additionally, in the LPPEHDWR system, the absence of the α-helix at residues 738–748 was responsible for enzyme inactivation. Moreover, the size of the substrate channel and cavity volume is significantly reduced after the binding of the inhibitory peptide to the protein, which is an important factor in the inhibition of enzyme activity. Moreover, IPI, by stabilizing the active site of DPP4, induces the disappearance of the horizontal α-helix and a notable reduction in the active cavity volume.

In summary, this study elucidates the inhibition mechanism of silkworm pupa peptide on the DPP4 enzyme, providing a significant theoretical basis for the advancement of related health foods and pharmaceuticals.

## Figures and Tables

**Table 1:** Probability of α-helix.

System	Residues 197–207	Residues 738–747
Apo	99.78%	87.81%
IPI	38.41%	94.58%
LPAVTIR	80.34%	91.10%
LPPEHDWR	6.68%	57.82%

**Table 2:** The cavity volume of the four systems.

Structure	Cavity Volume (Å^3^) (Apo)	Cavity Volume (Å^3^) (IPI)	Cavity Volume (Å^3^) (LPAVTIR)	Cavity Volume (Å^3^) (LPPEHDWR)
1	11,147.29	12,104.116	10,964.56	9702.332
2	11,883.58	11,105.567	12,076.32	10,092.49
3	11,223.88	10,284.191	11,278.85	11,852.07
4	12,424.45	11,679.295	12,636.11	11,189.88
5	11,050.15	10,975.048	10,643.82	10,714.59
6	11,979.49	12,231.661	10,834.91	11,675.83
7	11,090.71	9507.133	10,792.91	11,934.00
8	11,018.13	11,118.331	10,538.31	10,822.76
9	11,439.93	10,604.945	10,551.95	11,704.74
10	11,848.75	11,723.584	12,437.79	11,103.90
avg	11,510.64	11,133.3871	11,275.55	11,079.26
Standard Deviation	489.73	847.61	805.07	756.91

**Table 3:** The side and the bottom opening radius.

System	Side Opening Radius (Å)	Bottom Opening Radius (Å)
Apo	6.11	4.06
IPI	3.17	4.31
LPAVTIR	5.11	4.31
LPPEHDWR	4.31	4.04

**Table 4:** MM-PBSA (KJ/mol) of the two systems.

System	IPI	LPAVTIR	LPPEHDWR
ΔE_vdw_	−20.42 ± 0.59	−48.22 ± 0.66	−44.40 ± 0.83
ΔE_ele_	−158.48 ± 4.18	−413.84 ± 6.71	−119.86 ± 3.74
ΔG_solv_	167.69 ± 4.19	428.32 ± 6.05	131.35 ± 3.76
ΔG_gas_	−178.90 ± 4.47	−462.06 ± 6.48	−164.26 ± 4.27
ΔG_total_	−11.20 ± 0.72	−33.74 ± 0.79	−32.91 ± 0.72

## Data Availability

Data are contained within the article and supplementary materials.

## Supplementary Materials

The following supporting information can be downloaded at: https://www.mdpi.com/article/10.3390/ijms25020839/s1.
