# Supplementary material for: Study on the Mechanism of Interaction between Dipeptidyl Peptidase 4 and Inhibitory Peptides Based on Gaussian Accelerated Molecular Dynamic Simulation
Source: Int J Mol Sci. 2024 Jan 10;25(2):839. doi: 10.3390/ijms25020839 (PMC10815451; doi:10.3390/ijms25020839)
Supplement: Supplementary file 1 [file ijms-25-00839-s001.zip › ijms-2763846-supplementary.pdf]

Supplementary Materials

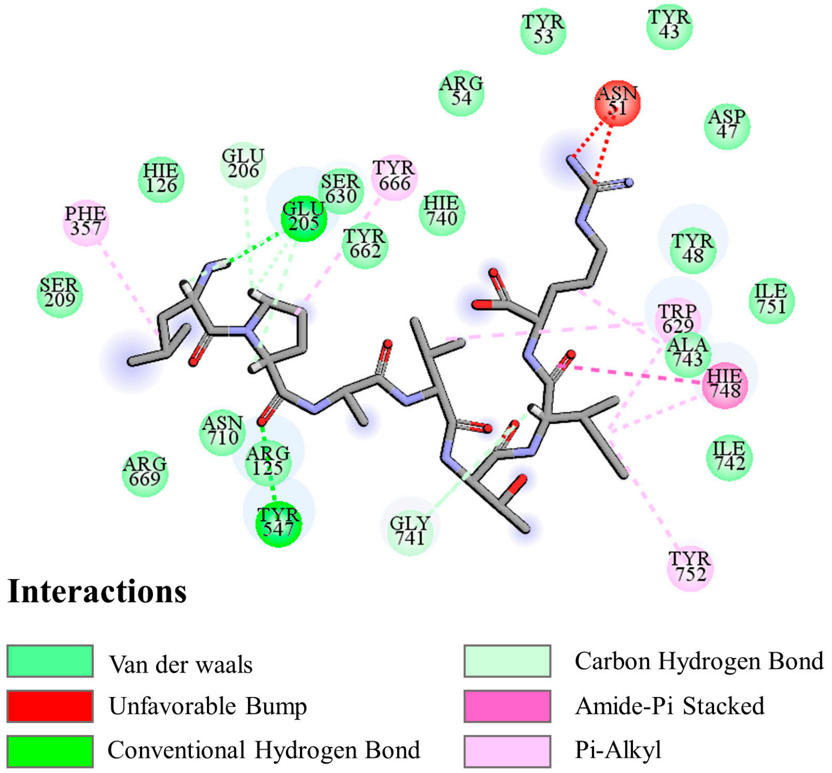

Figure S1. The active residues around the inhibitory peptide LPAVTIR binding to DPP4.

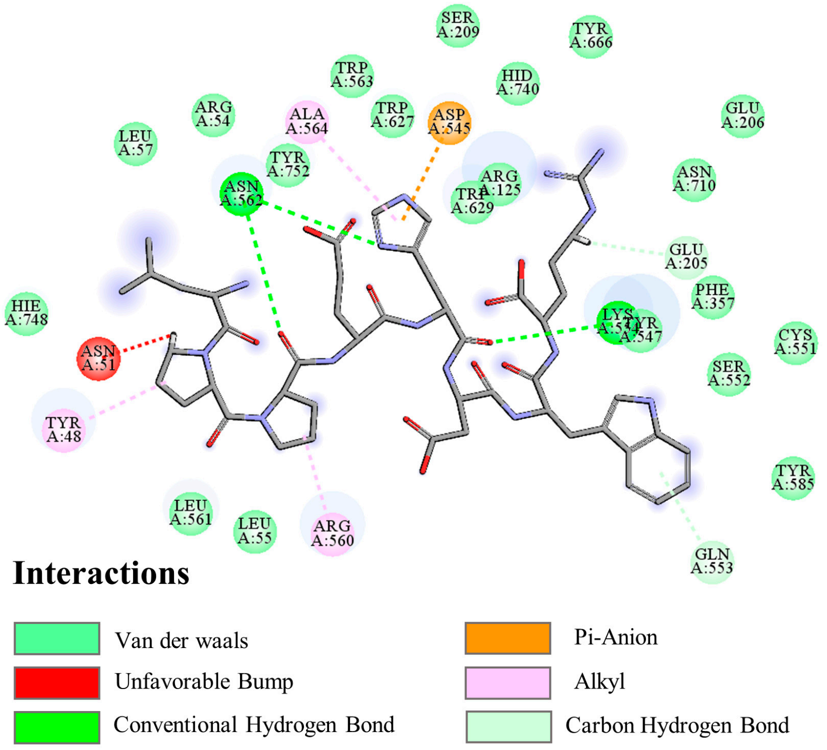

Figure S2. The active residues around the inhibitory peptide LPPEHDWR binding to DPP4.

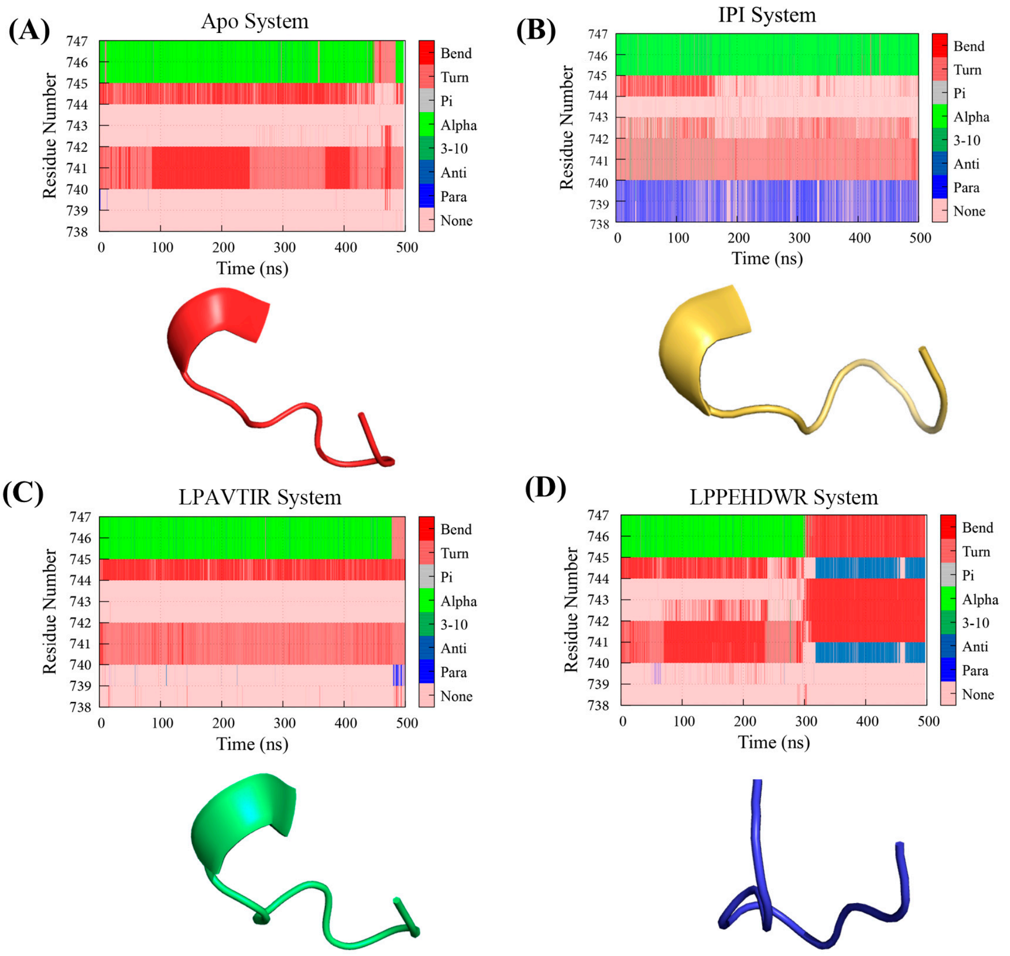

Figure S3. (**A**) The secondary structure changes' probability and the corresponding 3D structure changes of the Apo system in residues 738–747. (**B**) The secondary structure changes' probability and the corresponding 3D structure changes of the IPI system in residues 738–747. (**C**) The secondary structure changes' probability and the corresponding 3D structure changes of the LPAVTIR system in residues 738–747. (**D**) The secondary structure changes' probability and the corresponding 3D structure changes of the LPPEHDWR system in residues 738–747.
